# Inhibiting Voltage Decay in Li-Rich Layered Oxide Cathode: From O3-Type to O2-Type Structural Design

**DOI:** 10.1007/s40820-024-01473-7

**Published:** 2024-08-01

**Authors:** Guohua Zhang, Xiaohui Wen, Yuheng Gao, Renyuan Zhang, Yunhui Huang

**Affiliations:** 1 Institute of New Energy for Vehicles, Shanghai Key Laboratory for R&D and Application of Metallic Functional Materials, School of Materials Science and Engineering, Tongji University, Shanghai, 201804 People’s Republic of China; 2 Contemporary Amperex Technology Co., Ltd, Ningde, 352100 People’s Republic of China; 3 State Key Laboratory of Material Processing and Die and Mould Technology, School of Materials Science and Engineering, Huazhong University of Science and Technology, Wuhan, 430074 Hubei People’s Republic of China

**Keywords:** Lithium-ion batteries, Li-rich layered oxide, Voltage decay, Migration of transition metal ions, O2-type structural design

## Abstract

Li-rich layered oxide (LRLO) cathodes have been regarded as promising candidates for next-generation Li-ion batteries due to their exceptionally high energy density, which combines cationic and anionic redox activities. However, continuous voltage decay during cycling remains the primary obstacle for practical applications, which has yet to be fundamentally addressed. It is widely acknowledged that voltage decay originates from the irreversible migration of transition metal ions, which usually further exacerbates structural evolution and aggravates the irreversible oxygen redox reactions. Recently, constructing O2-type structure has been considered one of the most promising approaches for inhibiting voltage decay. In this review, the relationship between voltage decay and structural evolution is systematically elucidated. Strategies to suppress voltage decay are systematically summarized. Additionally, the design of O2-type structure and the corresponding mechanism of suppressing voltage decay are comprehensively discussed. Unfortunately, the reported O2-type LRLO cathodes still exhibit partially disordered structure with extended cycles. Herein, the factors that may cause the irreversible transition metal migrations in O2-type LRLO materials are also explored, while the perspectives and challenges for designing high-performance O2-type LRLO cathodes without voltage decay are proposed.

## Highlights

- This review systematically compares the different effects of O2-type and O3-type structures on voltage decay for Li-rich layered oxide (LRLO) cathode.
- The development of O2-type materials and the corresponding mechanisms for addressing voltage decay are comprehensively reviewed.
- The perspectives and challenges for designing high-performance O2-type LRLO cathodes without voltage decay are proposed.

## Graphical abstract

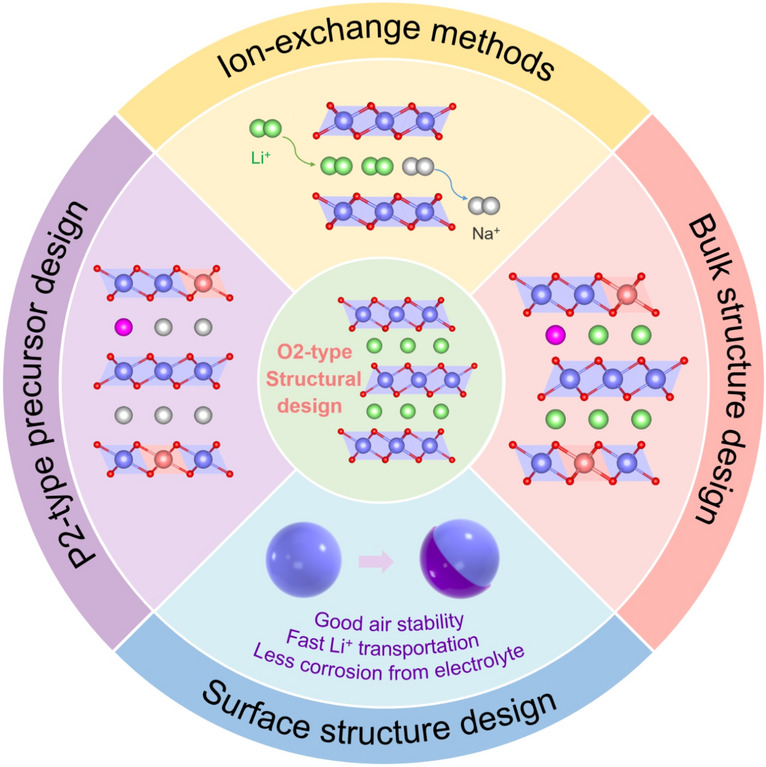

## Introduction

With the advent of electrified transportation, there is a pressing demand for improvements in rechargeable lithium-ion batteries. In particular, the energy density ceiling placed on the cathode materials has been a primary factor precluding the full-scale deployment of green energy technologies [1–3]. Unlike traditional cathode materials (layered LiCoO_2_, olivine LiFePO_4_, and Ni-rich materials), Li-rich layered oxide (LRLO) materials can deliver high discharge capacities over 250 mAh g^−1^ based on anionic/cationic redox chemistry, which have been regarded as promising candidates for next-generation Li-ion batteries [4]. However, LRLO materials suffer from detrimental voltage decay during cycling, resulting in continuous energy loss and increased complexity and difficulty in battery management systems, which seriously hindered its commercial application. Until now, the problem of voltage decay still has not been fundamentally solved. It is widely believed that the origin of voltage decay is mainly the irreversible migration of transition metal (TM) ions. Once the irreversible migration occurs, the phase evolution, lattice oxygen release, nanovoids, and TM densification may be triggered, finally resulting in the irreversible oxygen redox (OR) reaction and voltage decay [5–9].

For conventional O3-type LRLO materials, once TM ions migrate to the tetrahedral position between TM layer and Li layer, they can easily move and become trapped in neighboring octahedral Li sites due to the octahedral position of Li layer has a lower energy barrier [10, 11]. Excessive TM ions occupying the Li layer lead to the irreversible phase transformation from layered phase to spinel and rock salt phases. This affects the ability of the lithium ions to be deintercalated, resulting in voltage decay rapidly [12]. Conventional modification strategies, such as doping and surface coating, play a role in stabilizing the surface structure of LRLO and inhibiting side reactions with electrolyte. However, they cannot effectively overcome the challenge of irreversible migration. Given the challenges associated with inhibiting spontaneous cation migration in O3-type layered oxide cathodes, researchers have turned their attention to O2-type LRLO materials to address this issue. In the O2-type structure, TM ions find it difficult to migrate from the intermediate tetrahedral site into the adjacent octahedral Li site because of the high electrostatic repulsion between the face-share cations. This could facilitate the return of TM ions back to the original TM layer [1, 10, 11]. Therefore, tuning oxygen stacking arrangements and the coordination environment of alkali metal to construct an O2-type structure has been regarded as the most promising strategy to inhibit voltage decay [1, 13, 14].

In this review, we systematically elucidate the relationship between voltage decay and structural evolution (mainly including electronic structure, local structure, and phase transformations). We also summarize the strategies to inhibit the voltage decay. In addition, the design of O2-type LRLO materials and the corresponding mechanism for addressing voltage decay are comprehensively reviewed. The factors that may instigate the irreversible migration of TM ions in the O2-type structure are also discussed. Finally, the perspectives and challenges on the design of the O2-type LRLO materials without voltage decay are proposed.

## Mechanism of Voltage Decay

### Origin of High Capacity

**Fig. 1:**
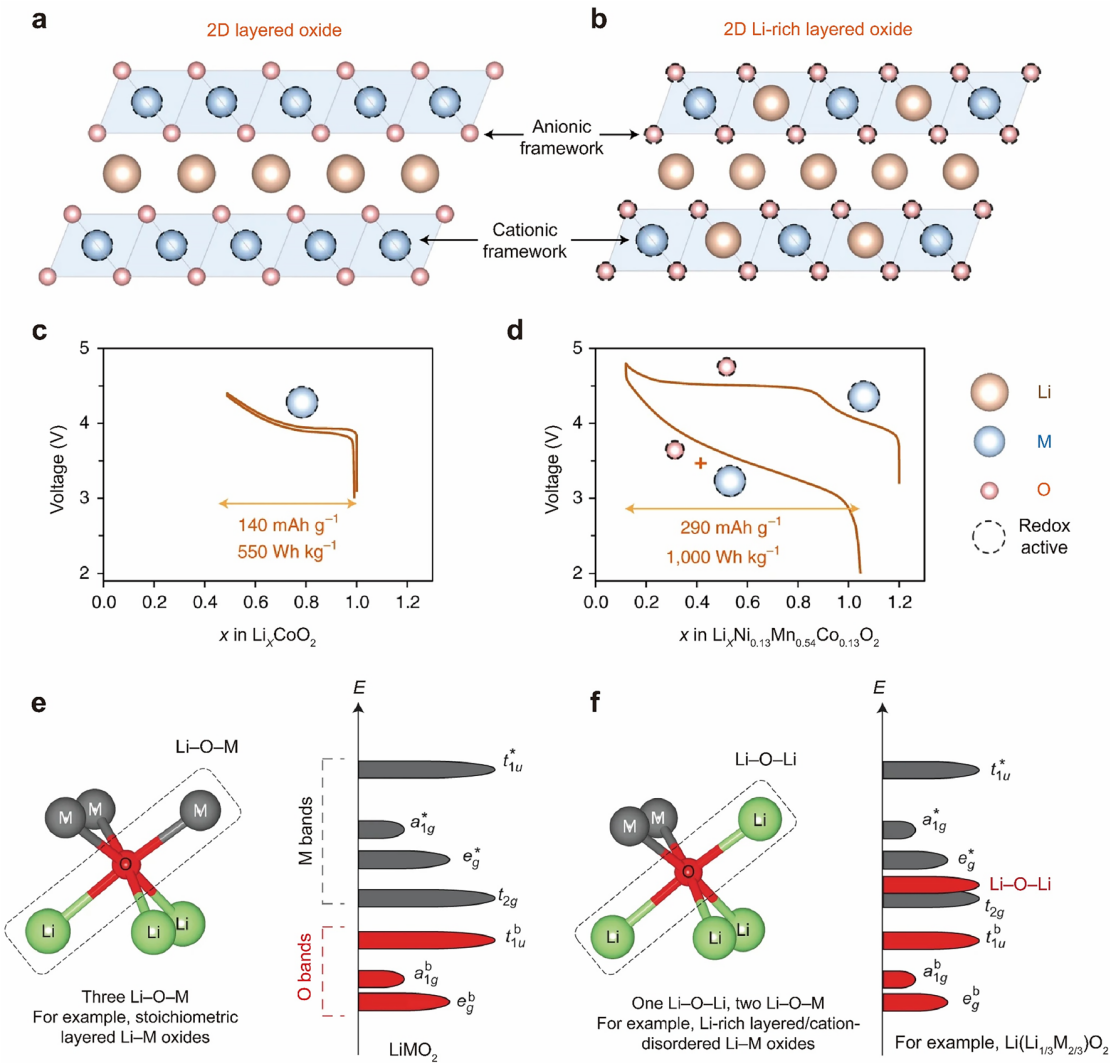
**a** Structure of layered oxides. **b** Structure of LRLO materials containing extra Li within the metal layers. **c, d** The corresponding voltage profiles indicate a near-doubling of capacity and specific energy for LRLO materials due to cumulative cationic and anionic redox processes. Reproduced with permission from Ref. [15]. Copyright 2018, Nature Publishing Group. **e** Local atomic coordination around oxygen consisting of three Li–O–M configurations in layered oxides and the corresponding schematic of the band structure. **f** Local atomic coordination around oxygen with one Li–O–Li and two Li–O–M configurations in LRLO materials and the corresponding schematic of the band structure. The Li–O–Li configurations lead to unhybridized O 2*p* states (Li–O–Li states) and as a result are more easily oxidized. Reproduced with permission from Ref. [20]. Copyright 2018, Nature Publishing Group

It has been widely accepted that oxygen redox reactions are responsible for the excess capacity beyond cation redox in LRLO materials (Fig. 1a–d) [2, 15]. Understanding the origin of high capacity is essential to deeply explore the relationship among irreversible TM migration, voltage decay, and irreversible oxygen redox in LRLO materials. Based on the development of advanced characterization technology, the mystery of the process of oxygen redox is gradually being revealed. In 2013, Sathiya et al. first confirmed the existence of lattice oxygen redox reactions and peroxo/superoxo-like species based on Li_2_Ru_1−*y*_Sn_*y*_O_3_ model materials [16]. In 2015, they further demonstrated the formation of reversible O_2_^*n−*^ species in Li_2_Ru_0.75_Sn_0.25_O_2_ [17], as well as the presence of O–O dimers (shortened O–O bonds) in the charged Li_2_IrO_3_ [18]. The observed bond length of the O–O dimer arising from the oxidation of lattice oxygen is approximately 2.5 Å, much longer than that in Li_2_O_2_ (1.5 Å), and thus it was named ‘peroxo-like’ species. In 2016, Luo et al. proposed an oxygen redox model in which the removal of Li^+^ is compensated by the formation of localized electron holes on O atoms coordinated by Mn^4+^ and Li^+^ ions. The process promotes electron–hole localization on O, rather than the formation of true O_2_^2−^ (peroxide, O–O ∼ 1.45 Å) species. The model recognizes the specific role of Mn and determines the fine balance between oxygen loss and oxygen redox chemistry [19]. In the same year, Seo et al. demonstrated that the oxygen redox activity originated from specific Li–O–Li configurations, in which orphaned oxygen states are lifted out of the bonding oxygen manifold and positioned in the TM-dominated complex of e_g_* and t_2g_ states. This leads to the competition between oxygen oxidation and TM oxidation (Fig. 1e, f) [20]. Note that it is also sometimes called ‘orphaned’ or ‘unhybridized’ O 2*p* state, or ‘O lone-pair’, or ‘Li–O–Li’ configuration, or the ‘b_1_* state’ in C_2*v*_ point-group symmetry [19–21]. This molecular orbital theory of Li–O–Li was further developed to include partially reversible out-of-plane TM migration to explain the shifting O-redox potential between charge and discharge [21]. The *d*-*d* Coulomb interaction term *U* was usually used to characterize the on-site electron repulsion within the *d* orbitals [22–24]. In 2018, Assat and Tarascon explored the reversible and stable anionic redox activity in different scenarios [15]. For highly ionic metal-ligand bonds, electrons are exchanged from the filled lower Hubbard bands. In highly correlated systems, the electrons directly removed from the non-bonding O 2*p* band, leading to partially irreversible processes. When the lower Hubbard bands and O 2*p* non-bonding bands overlap, it results in the reversible redox of oxygen [19, 25]. In 2021, Kitchaev et al. proposed that delocalized metal-oxygen *π*‑redox is the origin of anomalous nonhysteretic capacity [26]. They derived a consistent mechanism of nonhysteretic oxidation beyond the TM limit, explaining the electrochemical and structural evolution of the model materials. This work established a framework for engineering materials with reversible capacity exceeding TM redox.

Although a definitive consensus on the explanation of the oxygen redox process remains elusive, there is a prevailing agreement among these mechanisms. Specifically, the local structure of TMO_6_ octahedrons changes significantly in the oxygen redox process, mainly attributed to the irreversible migration of TM ions. Therefore, it is widely recognized that voltage decay is closely associated with the irreversible migration of TM ions.

### Origin of Voltage Decay

Due to the complexity of the structure of LRLO materials, it still remains challenging to achieve a unified understanding of the voltage fade mechanism. Currently, several prevailing theories have been established, including TM valence state reduction, irreversible TM migrations, irreversible phase transitions, oxygen release, multi-scale defects such as dislocation and oxygen pore [4–6, 27–31].

**Fig. 2:**
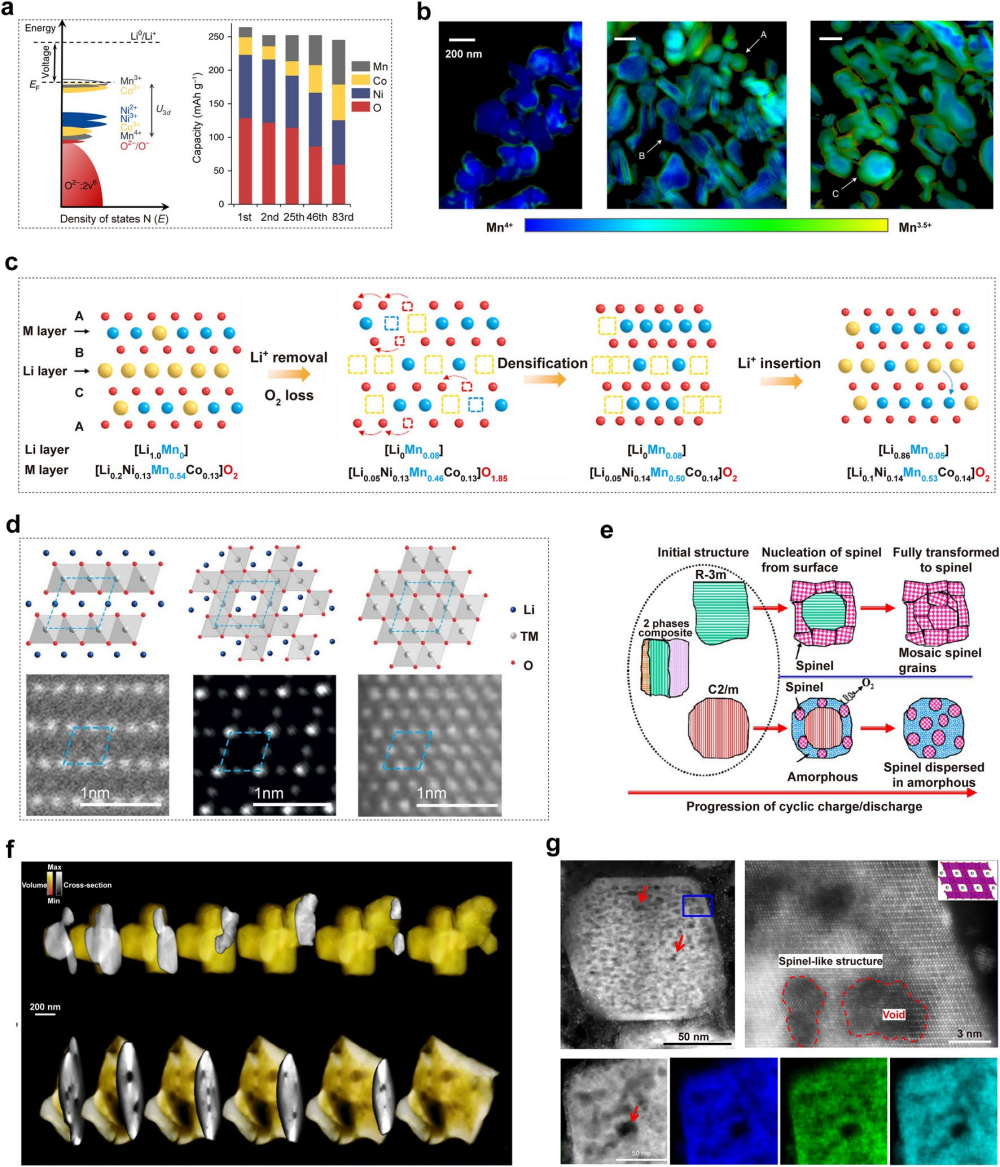
**a** Redox couple evolution of Li_1.2_Ni_0.15_Co_0.1_Mn_0.55_O_2_ during cycling involving a shift in the Fermi level and varying contributions from different elements. Reproduced with permission from Ref. [32]. Copyright 2018, Nature Publishing Group. **b** X-ray ptychography images of the pristine Li_1.18_Mn_0.53_Ni_0.21_Co_0.08_O_2_ material and the material after 1 cycle and 125 cycles. Reproduced with permission from Ref. [33]. Copyright 2021, Nature Publishing Group. **c** Schematic of the A  →  A′  →  P′ phase transition for Li_1.2_Ni_0.13_Co_0.13_Mn_0.54_O_2_ electrode during first cycling. Reproduced with permission from Ref. [7]. Copyright 2020, Nature Publishing Group. **d** Configurations of the layered, spinel, and rock salt phases. Reproduced with permission from Ref. [5]. Copyright 2022, American Chemical Society. **e** Schematic drawing showing that the initial LRLO material is composed of three phases: *R*-3*m*, *C*2/*m*, and nanocomposite of intergrowth of *R*-3*m* and *C*2/*m*. Reproduced with permission from Ref. [34]. Copyright 2013, American Chemical Society. **f** 3D electron tomography reconstruction of the pristine state and after 15 cycles Li_1.2_Ni_0.15_Co_0.1_Mn_0.55_O_2_ materials. Reproduced with permission from Ref. [32]. Copyright 2018, Nature Publishing Group. **g** STEM-HAADF image and EDS elemental maps of a Li_1.2_Ni_0.2_Mn_0.6_O_2_ cathode after 300 cycles at low and high magnifications. Reproduced with permission from Ref. [8]. Copyright 2019, Nature Publishing Group

Hu et al. have presented a novel insight into the origin of the voltage fading during cycling, utilizing Li_1.2_Ni_0.15_Co_0.1_Mn_0.55_O_2_ as the prototype material [32]. Leveraging a multi-length-scale X-ray spectroscopic technique, they observed a significant decrease in the discharge capacity contribution from oxygen and nickel redox couples after 83 cycles, while the contribution from manganese and cobalt redox couples showed a steady increase (Fig. 2a). Meanwhile, the reduction of manganese and cobalt activates the Mn^3+^/Mn^4+^and Co^2+^/Co^3+^ redox couples, elevating the Fermi level and reducing the open-circuit voltage and working voltage. Moreover, the reduction in TM elements weakens the covalent nature of M–O bonds, resulting in reduced oxygen participation in subsequent redox reactions. The transition from nickel/oxygen to manganese/cobalt redox couples was identified as the primary cause of the gradual voltage decay. In a related study, Csernica et al. demonstrated the progressive reduction of manganese and cobalt from their initial Mn^4+^ and Co^3+^ states through transmission-based X-ray absorption spectromicroscopy and ptychography on mechanically cross-sectioned Li_1.18−*x*_Ni_0.21_Mn_0.53_Co_0.08_O_2−*δ*_ electrodes (Fig. 2b) [33].

Microscopic strain has also been widely discussed as the origin of voltage decay [6]. The LRLO lattice comprises two domains: LiTMO_2_ and Li_2_MnO_3_. During the delithiation process, the LiTMO_2_ domain exhibits primary delithiation followed by the Li_2_MnO_3_ domain. Delithiation activation in the LiTMO_2_ domain induces an increase in local electrostatic repulsion force and lattice expansion. When the Li_2_MnO_3_ domain was activated, its lattice expansion is partially constrained, resulting in severe microscopic strain. The onset of lattice strain occurs at the particle surface and gradually propagates into its interior, peaking when the denudation of the LiTMO_2_ domain nears completion. These extreme strains significantly compromise structural stability, promote internal faults within LRLO, elevate energy barriers for Li^+^ reinsertion, and trigger oxygen release decomposition from Li_2_MnO_3_, thus contributing to voltage decay.

Bulk oxygen vacancies play a crucial role as an atomistic bridge, connecting the phenomenon of single-phase cation disordering and oxygen release, both of which progressively occur during cycling and have been independently proposed as contributing factors to voltage decay [11, 32]. In 2020, Tarascon et al. extended the cycling potential window for the Li_1.2_Ni_0.13_Co_0.13_Mn_0.54_O_2_ electrode, elucidating a novel mechanism of structural evolution. This mechanism involves the formation of a structurally densified single-phase A’ under harsh oxidizing conditions throughout the crystallites, not just at the surface [7]. It is consistent with the “lattice densification” model proposed by Delmas [35]. In the first electrochemical cycle, delithiation and oxygen evolution lead to the creation of vacancies in both the cationic and anionic sublattices upon charge to above 4.6 V (Fig. 2c). The migration of oxygen vacancies from the bulk to the surface is observed, followed by their subsequent annihilation as they are refilled. Additionally, the vacant Li sites are partially refilled with TM cations, leading to their presence in the octahedral interlayer sites and the suppression of the “honeycomb” Li-TM cation ordering. This phenomenon results in an increase in the TM/O atomic ratio, which is referred to as “densification” and aligns with findings from Boulineau’s research [9]. Subsequent discharge leads to the partial movement of TM cations back to their original positions, and the vacant cationic sites are filled with Li^+^. These findings offer valuable insights into the impact of irreversible TM migrations on structural evolution.

The presence of high-density dislocations also significantly impacts the voltage decay and oxygen activity of LRLO [31]. The nucleation of line defects disrupts the order of oxygen layers, leading to a notable alteration in the local lithium environment. For instance, in the highly delithiated state, the initial stacking sequence ABCABC is transformed into ABCBCABC, which includes a partial BCBC similar to O1. In the most energetically favorable O3 structures, all Li octahedral positions share edges with transition metal octahedral positions. However, transitioning partially to O1 results in unfavorable surface sharing between Li and TM octahedral positions, thereby increasing the Gibbs free energy of the system. It is plausible that Class O1 defects introduce a free energy difference that contributes to voltage decay. After annealing at elevated temperatures, voltage recovery is restored along with stacking order, further substantiating the correlation between oxygen accumulation order and voltage decay.

The process of oxygen release is accompanied by the transfer of electrons from O^*n−*^ ions to TM ions, resulting in the reduction and migration of TM ions. Therefore, oxygen release is also considered to be a significant cause of voltage decay [36]. Zhou et al. illustrated the degradation pathway associated with phase transformation during oxygen release: layered phase → spinel phase → rock salt phase, as shown in Fig. 2d. The formation of oxygen vacancies is implied by the concurrent oxygen release during the layered-to-spinel phase transition. With further oxygen loss, the increasing number of oxygen vacancies aggravates the transformation from the layered phase to the rock salt phase. In 2013, Gu et al. investigated the phase evolution behavior of LRLO during cycling and found that the migration of TM cations into the Li layer initiates the transformation to spinel for the *R*3*m* phase region during cycling, as depicted in Fig. 2e [34]. Conversely, under high-voltage cycling conditions, the Li_2_MnO_3_ phase exhibits amorphous and disordered characteristics due to oxygen release and structural degradation, leading to crystal lattices breakdown. STEM imaging and EDS mapping provide evidence for the layered-to-spinel transformation, lattice distortion/amorphization, crack formation, and porosity development. Notably, the nucleation and growth mechanism governs the phase transformation of layered-to-spinel, resulting in spinel clusters with diverse orientations within distorted/amorphized lattices, significantly impacting structural stability and energy density.

Oxygen pores have been identified as an additional cause of voltage decay. When the defect density of the bulk phase oxygen vacancy is excessively high, it can lead to the loss of multiple coordinated anionic oxygen atoms in the coordination of a transition metal resulting in the migration or dissolution of TM and the release of other coordination oxygen in the form of oxygen molecule. This process creates a hole in the material, which is referred to as an oxygen pore in the literature. In 2018, Hu et al. discovered numerous pores encased in a thin Mn^2+^ shell within the bulk of the cycled Li_1.2_Ni_0.15_Co_0.1_Mn_0.55_O_2_ electrode using 3D electron tomography reconstruction based on atomic-resolution annular dark-field STEM (ADF-STEM) imaging, spatially resolved electron energy loss spectroscopy (EELS) and ADF-STEM tomography (Fig. 2f). They proposed that these pores formed due to oxygen release and related lattice densification, suggesting that densification occurs both on the surface and within the bulk of LRLO. In 2019, Yan et al. observed that defects originating on the particle surface are continuously transferred into the bulk lattice by the same reconstruction techniques (Fig. 2g) [8]. This bulk degradation involves nanovoid formation and lattice structure transformation, resulting from electrochemically driven oxygen vacancy formation at particle surfaces and their subsequent migration toward the bulk lattice through a condensation process. This sharply contrasted with thermally driven processes. This process is associated with a high cutoff voltage that activates an anionic redox. First-principle calculations reveal that this redox process significantly reduces both the formation energy of oxygen vacancies and the migration barrier of oxidized oxide ions, thus facilitating the migration of oxygen vacancies into the bulk lattice of the cathode. The progressive surface-to-bulk degradation underscores the importance of suppressing oxygen vacancy generation at the particle surface to prevent further injection of vacancies and enhance the structural stability of LRLO materials.

The voltage decay mechanism in LRLO materials involves a multifaceted process that begins with the high-voltage-triggered oxidation of lattice oxygen, a crucial step enabling these materials to achieve their ultra-high capacity. This process induces substantial anisotropic changes in the M–O bonds, accompanied by the irreversible TM migration from the TM layer to the Li layer. This irreversible migration is the primary cause of various subsequent phenomena such as oxygen release, phase transitions, oxygen vacancies, voids, densification, and more, collectively contributing to voltage decay. Therefore, the TM migration can be regarded as the origin of voltage decay. It is believed that inhibiting this irreversible migration is the most effective method to overcome voltage decay without sacrificing the capacity of LRLO materials.

## Strategies to Inhibit Voltage Decay

### Traditional Strategies to Mitigate Voltage Decay

**Fig. 3:**
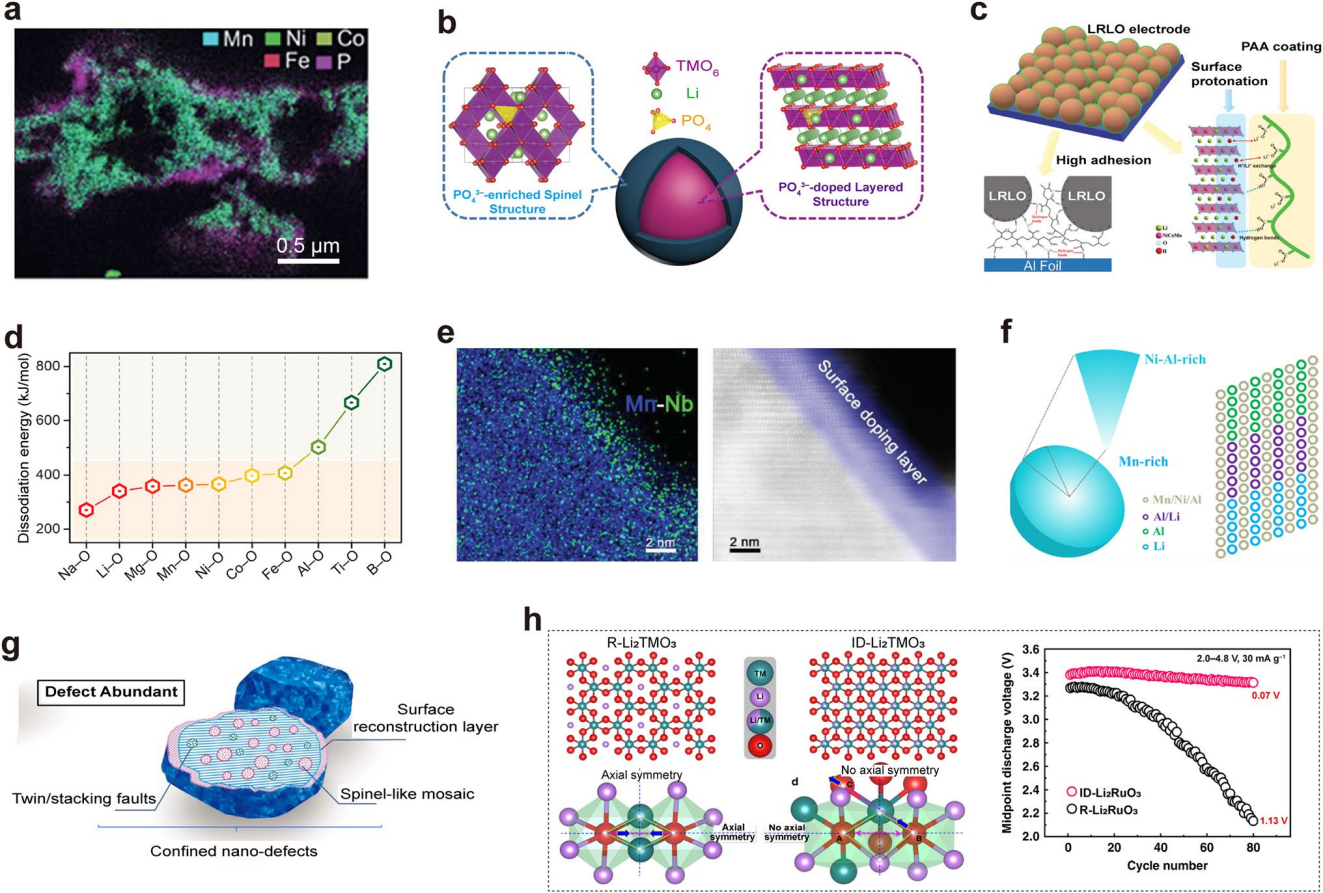
**a** Cross-sectional SEM–EDS image of a LiFePO_4_ coating Li_1.2_Ni_0.13_Co_0.13_Mn_0.54_O_2_ and corresponding EDS elemental mapping of O, P, Mn, Fe, Co, and Ni. Reproduced with permission from Ref. [38]. Copyright 2015, Wiley–VCH. **b** Schematic illustration of surface structural transition induced by gradient polyanion doping in Li_1.17_Mn_0.5_Ni_0.17_Co_0.16_O_2_ materials. Reproduced with permission from Ref. [39]. Copyright 2016, Wiley–VCH. **c** Multifunction protections of polyacrylic acid binder for Li_1.2_Ni_0.13_Co_0.13_Mn_0.54_O_2_ cathode: (1) Uniform coating layer to protect cathode particles; (2) H^+^/Li^+^ exchange to stabilize the crystal structure; (3) strong adhesive strength to keep the integrity of the electrode. Reproduced with permission from Ref. [40]. Copyright 2020, Wiley–VCH. **d** Element selection for surface doping. The dissociation energy of corresponding M–O bonds is vital for surface reinforcement. Reproduced with permission from Ref. [43]. Copyright 2021, Elsevier. **e** EDS mapping and HAADF image of the surface doping layer for Li_1.2_Ni_0.13_Co_0.13_Mn_0.54_O_2_ cathode. Reproduced with permission from Ref. [44]. Copyright 2018, Wiley–VCH. **f** Ni/Mn and Al dual concentration-gradients in Li_1.2_Mn_0.6_Ni_0.2_O_2_ assembled microspheres. Reproduced with permission from Ref. [47]. Copyright 2021, American Chemical Society. **g** Diagrams of the bulk-modified nanoscale defect-abundant Li_1.143_Mn_0.544_Ni_0.136_Co_0.136_O_2_ cathode. Reproduced with permission from Ref. [48]. Copyright 2019, Elsevier. **h** Crystal structures and structural response modes for the intralayer disordered (ID)-Li_2_RuO_3_ and regular (R)-Li_2_RuO_3_. Reproduced with permission from Ref. [49]. Copyright 2020, Nature Publishing Group

To address the voltage decay issue in LRLO cathodes, various strategies have been proposed. These strategies fall into two main categories: surface modification and bulk phase regulation. Surface modification techniques include surface coating/doping, morphological design, component modulation, and the modulation of binder/electrolyte. For example, the surface chemical treatment technology, like constructing the gradient Al doping and uniform LiAlO_2_/Li_3_PO_4_ protective layer on the surface of the layered oxide cathode materials to affect the stability of the bulk phase structure, is considered to be an effective method to suppress voltage decay [37]. Zheng et al. embedded nanoscale electrochemically active LiFePO_4_ into the surface of the Li_1.2_Ni_0.13_Co_0.13_Mn_0.54_O_2_ cathode by a sol–gel method, combining surface coating with bulk doping advantages (Fig. 3a) [38]. This nanoscale LiFePO_4_ layer effectively prevents side reactions between the cathode and organic electrolytes, promotes ion and charge transfer, restricts TM ion migration, and mitigates voltage fade during high-voltage cycling. Additionally, constructing a nanoscaled spinel-like surface layer with gradient polyanion doping strategy on Li_1.17_Mn_0.5_Ni_0.17_Co_0.16_O_2_ materials helps prevent continuous corrosion from organic electrolytes and facilitates Li^+^ and electron transport [39]. This approach involves doping a moderate amount of polyanions into the bulk material, which stabilizes the oxygen-packed structure and enhances the structural stability, thereby suppressing voltage decay. (Fig. 3b). Moreover, polyacrylic acid (PAA) has been utilized by Yang et al. as a multifunctional binder to stabilize the lattice structure of the Li_1.2_Ni_0.13_Co_0.13_Mn_0.54_O_2_ cathode, which exhibits only 1.04 mV voltage decay per cycle and 88% capacity retention over 500 cycles. (Fig. 3c) [40]. The structural stability is attributed to the reaction of carboxyl groups in PAA with surface oxygen species, forming a uniform and tight coating that significantly suppresses the dissolution of TM ions. Additionally, a proton-doped surface layer, formed by a H^+^/Li^+^ exchange reaction between the LRLO and PAA, efficiently alleviates TM ion migration, leading to lower voltage decay. Novel binders such as sodium carboxymethyl cellulose (CMC) and xanthan gum have also been explored to enhance the structural integrity and suppressed voltage decay of LRLO cathodes [41, 42]. Strengthening covalent bonds within the oxygen framework has been extensively studied (Fig. 3d). For example, Yin et al. proposed a surface reinforcement strategy by surface doping B to improve the structure stability and suppress the voltage decay of Li_1.144_Ni_0.134_Co_0.134_Mn_0.544_O_2_ [43]. As a result, the target material exhibited a suppressed voltage decay rate of 2.5 mV per cycle during the first 80 cycles at 0.2 C within 2.0–4.8 V. This work provides a new perspective to mitigate voltage decay by stabilizing surface oxygen framework of LRLO materials. Liu et al. introduced Nb^5+^ ions to the Li layer near the oxide surface, which helps inactivate surface oxygen and stabilize the structure of Li_1.2_Ni_0.13_Co_0.13_Mn_0.54_O_2_ cathode through strong Nb–O bonding (Fig. 3e**)** [44]. The Mn^4+^ ions remain inert both in the bulk and on the surface under various charge/discharge states, beneficial for avoiding distortion of the TMO_6_ octahedron and preventing the typical layered-to-spinel transformation. This strategy effectively minimized the average discharge potential drop per cycle, underscoring the critical role of inactivating surface oxygen in combating voltage decay. Other strategies like constructing integrated surface layers have also been proposed to improve both voltage and capacity stability [45, 46].

This chapter briefly reviews methods of bulk phase regulation, such as gradient doping, structural defects, and local structure control, which are employed to suppress voltage decay. Luo et al. designed dual concentration-gradients Li_1.2_Mn_0.6_Ni_0.2_O_2_ microspheres to suppress the voltage decay using a solvothermal method, based on solubility differences between NiCO_3_ and MnCO_3_ (Fig. 3f) [47]. The Mn-poor/Ni-rich surface and Al-rich surface effectively mitigated interfacial reactions, structural evolution, and the formation of lower-valence Mn^3+^/Mn^4+^ or Mn^2+^/Mn^3+^ redox couples during cycling. As a result, its voltage fading rate after 100 cycles is as small as 0.97 mV/cycle at 100 mA g^−1^ between 2.0 and 4.8 V. Guo et al. proposed a novel strategy to mitigate the detrimental voltage decay by incorporating abundant nanoscale defects into materials’ lattices, creating a bulk-modified Li_1.143_Mn_0.544_Ni_0.136_Co_0.136_O_2_ cathode through direct in-depth chemical delithiation (Fig. 3g) [48]. This modification featured multitudinous boundary-densities, reconstructed oxygen-deficient multi-phase surfaces, local spinel-like domains, twin-orderings and (or) stacking faults, leading to a slight voltage decay about 0.281 mV per cycle during 500 cycles. In addition, they engineered uniform oxygen vacancies via a gas–solid interface reaction to preserve structural integrity and address voltage decay in LRLO cathodes [50]. Ning et al. proposed a novel structural response mechanism that inhibits O–O dimerization by adjusting the local symmetry around oxygen ions, achieving negligible voltage decay (Fig. 3h) [49]. By disordering the TM/Li arrangement in the TM layer, the local symmetry exhibits a telescopic O–Ru–O configuration instead of O–O dimerization during the oxygen redox process, enhancing anionic redox stability, structural stability, and voltage stability. The critical role of tuning local symmetry in suppressing voltage decay in LRLO cathode materials was emphasized. Moreover, widening the gap bands between metallic and anionic and creating a Li-deficient pristine state also helped inhibit lattice oxygen escape and cation migration, resulting in low-voltage decay rate and prolonged cyclic stability [51, 52].

Nevertheless, these modification methods only partially mitigate voltage decay and improve electrochemical performance in O3-type LRLO materials. It is evident that the traditional strategies fall short in addressing the inherent flaws of the O3-type structure. In this structure, TM ions initially migrate to the nearest tetrahedral sites in the Li layer, and subsequently move to adjacent octahedral Li sites during cycling, inhibiting the reversible movement of TM ions back to their original sites [10, 11]. Thus, the O3-type structure struggles to effectively prevent the irreversible TM metal migrations, making it challenging to address voltage decay problem in O3-type LRLO materials. Fortunately, the O2-type structure has shown significant promise in inhibiting voltage decay by controlling the reversible migration of TM ions.

### Constructing O2-Type Structure to Suppress Voltage Decay

The irreversible TM migration is closely associated with the voltage decay, the lattice oxygen release, and structural evolutions. Although various modification methods have been developed to mitigate voltage decay and improve electrochemical performance of LRLO materials, achieving the performance standards necessary for industrial applications remains challenging. In response, researchers have focused on developing the O2-type structure as a promising alternative to address these issues. A comprehensive understanding of the O2-type configuration is essential for the design of high-performance LRLO cathode materials that can overcome voltage decay, a longstanding goal within both scientific and industrial communities. This involves a deep dive into the research history, design principle, and preparation method of O2-type layered oxide materials, which are crucial for the structural design of O2-type LRLO cathodes.

#### Research History of the O2-Type Structure

**Fig. 4:**
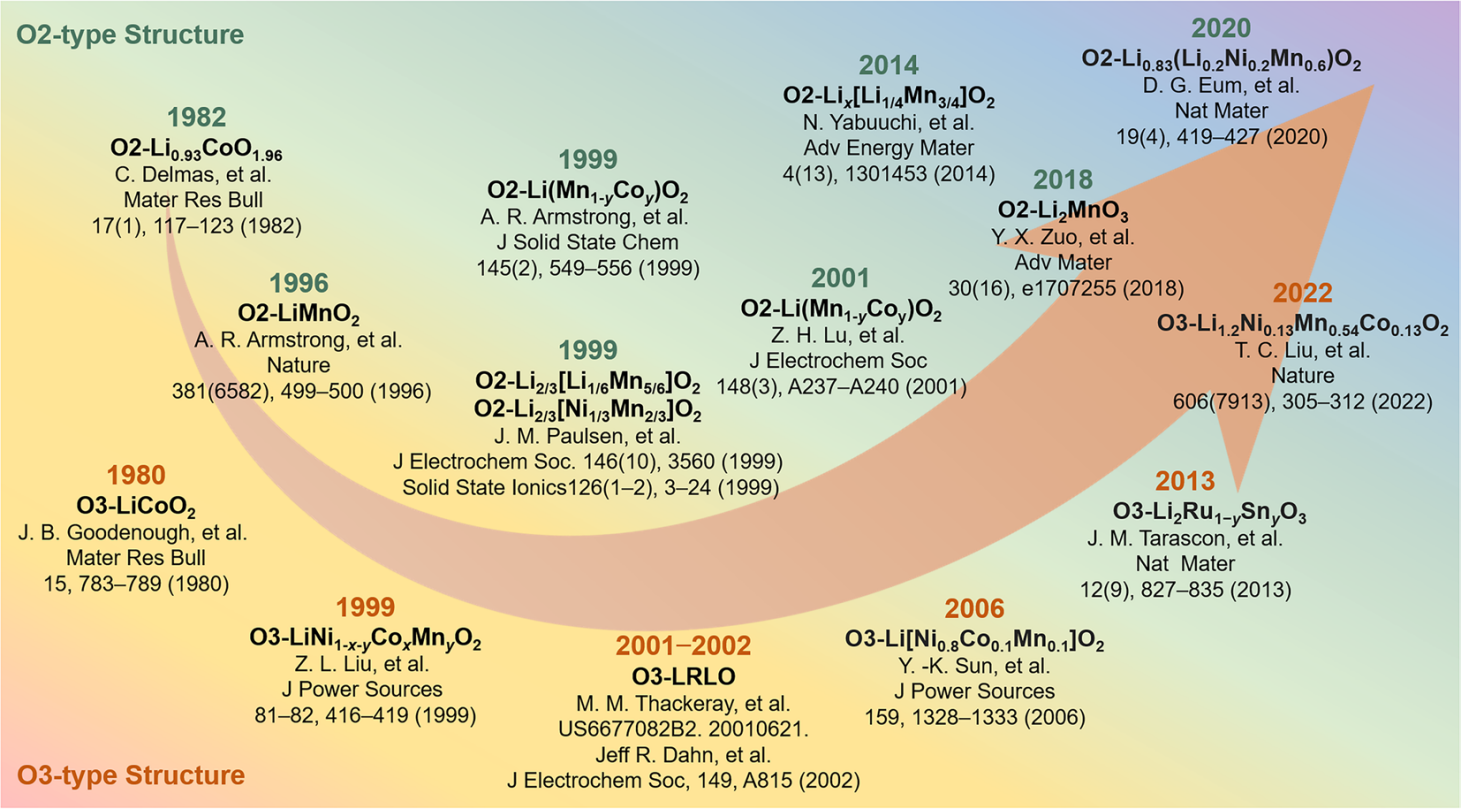
Research history of the O2-type and O3-type layered oxide materials

As shown in Fig. 4, the exploration of O2-type layered oxide materials began in 1982 when Delmas and colleagues synthesized a novel O2-type Li_0.93_CoO_1.96_ by heating in a methanol solution of lithium chloride, using Na_0.70_CoO_1.96_ as the starting material [53]. By 1984, they further explored the electrochemical (de)intercalation behaviors of O2-type Li_*x*_CoO_2_, a metastable structure that could transform into well-crystallized O3-type LiCoO_2_ upon heating [54, 55]. To better understanding, a series of unusual structures are experimentally observed during the process of lithium (de)insertion. They performed first-principles investigations on phase stability within the O2-LiCoO_2_ system and proposed mechanisms based on the *in situ* X-ray diffraction study [56]. The results suggest that O2 domain growth was faster than the formation of the nucleation centers and kinetically activated by a P2-Na_0.70_CoO_2_ → P2*-Na_~0.50_CoO_2_ phase transition [57]. In 2013, Li_2/3_Co_2/3_Mn_1/3_O_2_ was prepared by Na/Li ion exchange, and its thermal evolution was investigated by thermal analyses and X-ray diffraction [58]. More recently, in 2022, Xue et al. demonstrated that O2-type LiCoO_2_ cathodes exhibit significantly enhanced cycle performance in neutral aqueous electrolyte, attributing this improvement to the reduced structural degradation from electrophilic proton attacks [59].

The success of O2-type LiCoO_2_ led researchers to investigate layered LiMnO_2_ as an intercalation host for lithium-ion batteries, highlighting the benefits of layered structures for Li^+^ transport and the economic and environmental advantages of substituting cobalt with manganese. In 1996, based on Na/Li ion exchange, Bruce et al. prepared a new layered LiMnO_2_ compound analogous to LiCoO_2_ by refluxing NaMnO_2_ with an excess of LiCl or LiBr in *n*-hexanol at 145–150 °C for 6–8 h [60]. Subsequently, the structure of layered LiMnO_2_ electrodes was characterized by electron diffraction and lattice imaging techniques [61]. In the same year, they synthesized the layered Li(Mn_1−*y*_Co_*y*_)O_2_ solid solutions between LiMnO_2_ and LiCoO_2_ by the same method and investigated the effect of Co substitution on the structural stability and electrochemical performance [62, 63]. In addition, the influence of the ion exchange conditions on the structure and performance for the Li_*x*_(Mn_1−*y*_Co_*y*_)O_2_ electrodes was further investigated [64].

In 1999, Dahn et al. had synthesized O2-type Li_2/3_[Li_1/6_Mn_5/6_]O_2_ from P2-type Na_2/3_[Li_1/6_Mn_5/6_]O_2_ precursors using a similar ion exchange method, showing that these materials did not convert to spinel during cycling, unlike layered and orthorhombic LiMnO_2_ [65]. In the same year, the O2-type LiCoO_2_ and O2-type Li_2/3_[Ni_1/3_Mn_2/3_]O_2_ with well-ordered crystallinity were prepared [66]. Subsequently, the electrochemical properties, structural, and ion exchange reaction mechanism were comprehensively studied for O2-type Li_2/3_[Ni_1/3_Mn_2/3_]O_2_ materials [67, 68]. Moreover, they explored the intercalation of water in P2, T2, and O2 structure A_*z*_[Co_*x*_Ni_1/3−*x*_Mn_2/3_]O_2_. P2-Na_2/3_(H_2_O)_2/3_[Co_1/3_Mn_2/3_]O_2_ existed in the ideal P2 structure (*P*63/*mmc*) with the oxygen atoms of the water molecule located on the 2c site, while water could not be intercalated into the corresponding lithiated phases Li_2/3_[Co_*x*_Ni_1/3−*x*_Mn_2/3_]O_2_ [69]. The effect of Co substitution for Ni on the structure and the electrochemical behavior of Li_2/3_[Co_*x*_Ni_1/3−*x*_Mn_2/3_]O_2_ was investigated. Co additions could suppress the superlattice ordering of TM ions in the TM layers and destabilize the T2 structure [70].

#### Design Principle and Preparation Methods of the O2-Type Structure

**Fig. 5:**
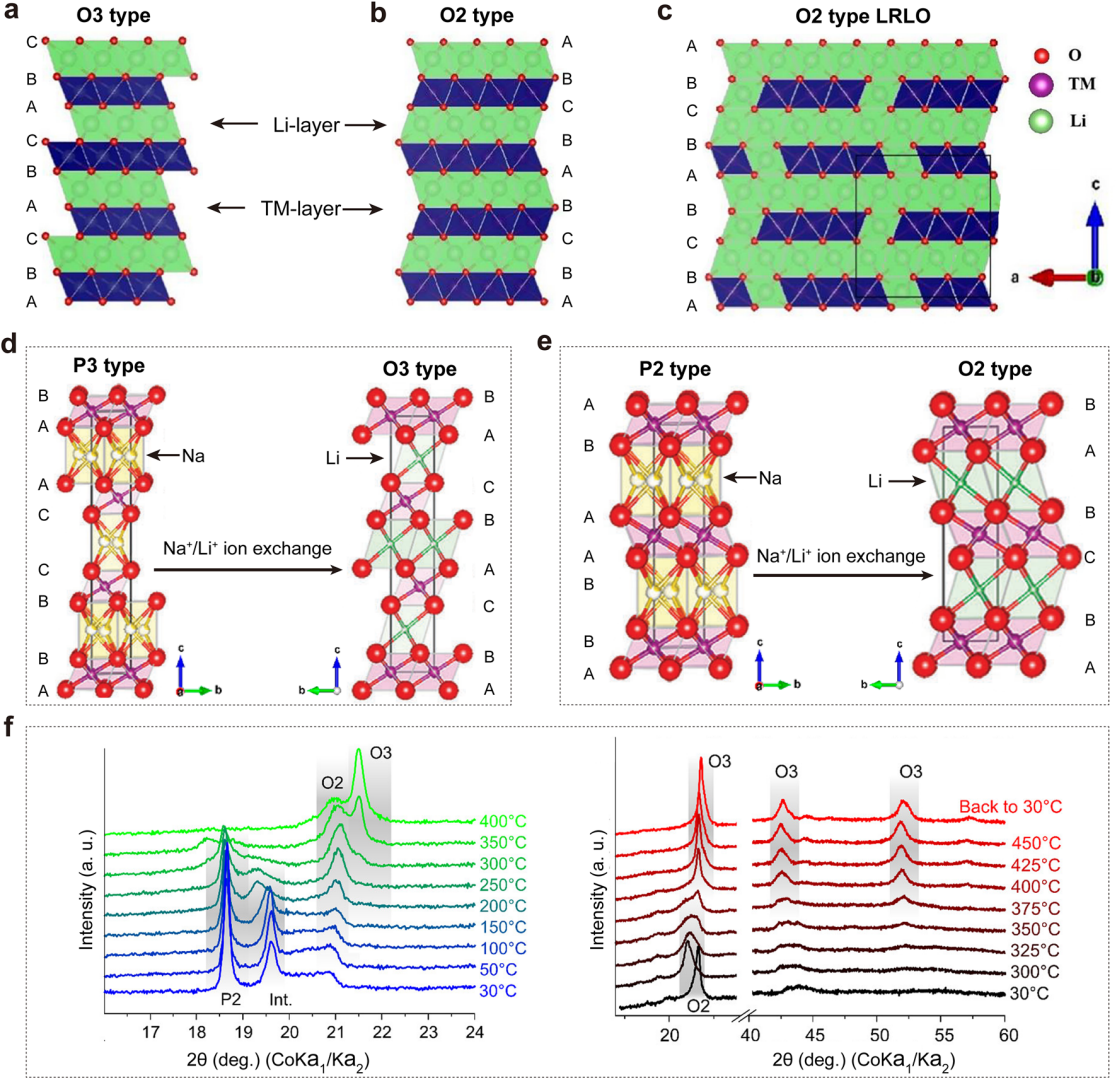
**a** Crystal models of O3-type structures. **b** Crystal models of O2-type structures. **c** A simulation model of O2-type LRLO material. Reproduced with permission from Ref. [72]. Copyright 2022, Elsevier. **d**, **e** Schematic illustration of ion exchange-induced phase transformation from P3-type and P2-type Na_2/3_(Ni_0.25_Mn_0.75_)O_2_ oxides to O3-type and O2-type Li_2/3_Ni_0.25_Mn_0.75_O_2_ oxides, respectively. Reproduced with permission from Ref. [74]. Copyright 2021, American Chemical Society. **f** Variable-temperature X-ray diffraction patterns recorded in a helium flow during the ion exchange reaction between P2-Na_0.7_[Li_0.14_Ni_0.14_Mn_0.72_]O_2_ and LiCl, and the irreversible structural transition from the O2-Li_0.84_Ni_0.14_Mn_0.72_O_2_ to the O3-Li_0.84_Ni_0.14_Mn_0.72_O_2_ phase. Reproduced with permission from Ref. [73]. Copyright 2023, American Chemical Society

According to the classification proposed by Delmas et al. based on the coordination environment of alkali metal ions and the oxygen stacking sequences, layered oxides are primarily classified into O2 (ABCBAB or ABACAB), O3 (ABCABC), P2 (ABBA), and P3 (ABBCCA) structures in Fig. 5a–e [53, 54, 71, 72]. In this classification, “O” and “P” represent octahedral and prismatic structure, respectively, while “2” or “3” means the number of MO_2_ layers in the unit cell. Sodium ions can occupy both prismatic and octahedral structures, whereas lithium ions are confined to octahedral structures. Most layered oxides materials possess an O3-type structure, with Li^+^ occupying octahedral sites in the interslab space and TMO_6_ octahedra sharing edges with adjacent LiO_6_ octahedra. Conversely, the O2-type structure features octahedral sites for Li that not only share edges with one TMO_6_ octahedron on one side, but also one face with another TMO_6_ octahedron on the other side [53, 73]. Typically, O3-type layered oxides are synthesized by conventional high-temperature solid-state methods or soft chemical routes (Fig. 5d). However, O2-type layered oxides, being metastable materials, cannot be achieved though high-temperature lithiation reactions but are instead obtained via ion exchange reactions using P2-type sodium layered oxides as precursors (Fig. 5e). This process preserves layered structure and oxygen stacking [53, 65].

Currently, almost all O2-type layered oxides were produced through chemical ion exchange methods, with the Na/Li ion exchange reaction being spontaneous and thermodynamically favored. Initially, P2-type precursors were dispersed in an organic solution or molten salts containing lithium compounds, such as LiCl, LiBr, and LiNO_3_ [67, 73, 75]. In an organic solution, the ion exchange process generally occurs under reflux or hydrothermal conditions, which require proper heating to enhance the rate of the ion exchange reaction. Afterward, the samples are washed and filtered multiple times to remove any residues from the exchange solution or salts, and then dried to obtain the target materials. For solid-state molten salts methods, the reaction temperature critically influences the ion exchange process. Delmas et al. investigated the impact of temperature on the structural evolution through variable-temperature X-ray diffraction from room temperature to 400 °C, revealing that the higher reaction temperature lead to a phase transformation from O2-type to O3-type structures (Fig. 5f). In addition, heating pure O2-type materials without the presence of LiCl salt underscored the importance of maintaining an appropriate reaction temperature to achieve the pure O2-type phase [54, 73]. This comprehensive overview of the research history, design principles, and preparation methods of O2-type layered oxide materials provides valuable insights and guidance for the design and preparation of O2-type LRLO materials.

#### Mechanism of the O2-Type Structure for Suppressing Voltage Decay

The origin of voltage decay in LRLO materials is widely attributed to the progressive structural evolution involving irreversible TM migration, which exacerbates the process of irreversible oxygen redox reaction and the release of lattice oxygen [1, 10, 11, 13, 14, 27, 76, 77]. For example, Li et al. demonstrated that in Li_1.2_Ni_0.2_Ru_0.6_O_2_ without oxygen activity, the direct cause of voltage decay was microstructural evolution, primarily originated from the irreversible TM migration [76]. Unfortunately, the spontaneous cation migration is inevitable due to the inherent structural properties. Traditional modification strategies, such as doping and surface coating, cannot fully eliminate the migration of TM ions. The design principles and preparation methods of O2-type layered oxide materials have been widely investigated, which provides an important foundation to explore the effects of O2-type structure on voltage decay in LRLO cathode materials.

**Fig. 6:**
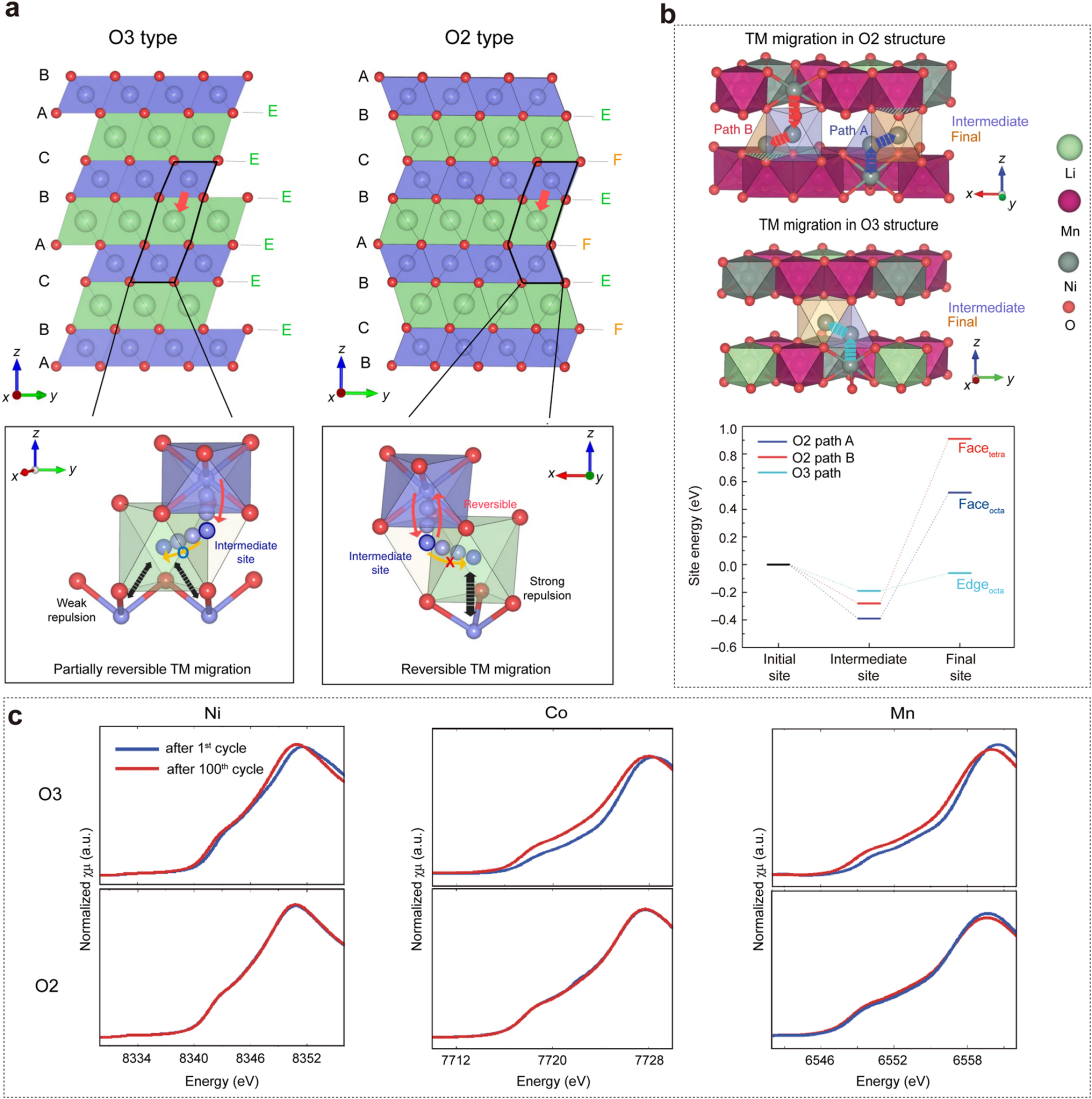
**a** Comparison of crystal structures and cation migration paths between O3-type and O2-type LRLO. **b** TM migration paths from initial to intermediate to final Li sites, and relative site energies of intermediate and final sites calculated along the migration paths of TM ions. Reproduced with permission from Ref. [1]. Copyright 2020, Nature Publishing Group. **c** Comparison of XANES spectra change during cycling for O3-type and O2-type Li_1.2_Ni_0.13_Co_0.13_Mn_0.54_O_2_. Reproduced with permission from Ref. [79]. Copyright 2020, American Chemical Society

In 2020, Eum et al. demonstrated that the O2-type structure could restrict the migration of TM ions within the lithium layer, effectively streamlining the return migration path of TM ions. This enhanced reversibility mitigates the asymmetry of the anionic redox in LRLO materials, promoting high-potential anionic reduction and decreasing the subsequent voltage hysteresis [1]. As illustrated in Fig. 6a, the local environments of Li sites differ significantly between O2-type and O3-type structures. In the O2-type structure, LiO_6_ octahedra in Li layer share faces with TMO_6_ octahedra in the TM layer, whereas in O3-type structure, they share only edges [65]. In O3-type LRLO materials, TM ions migrate to the nearest neighboring tetrahedral sites in the Li layer and then naturally move to adjacent octahedral Li sites due to thermodynamic preference, limiting the reversible return of TM ions [10, 11]. Conversely, in the O2-type structure, the migration from intermediate tetrahedral sites to adjacent octahedral Li sites is limited on account of the large electrostatic repulsion between cations in face-shared octahedral sites. This facilitates the return of migrated TM ions to the octahedral sites in the TM layers during discharge [1, 78]. The first-principles calculations further prove that TM migration from intermediate sites to adjacent octahedral sites requires overcoming a larger energy barrier in the O2-type structure compared to the O3-type structure (Fig. 6b). These findings highlight that regulating the reversibility of cation migration by constructing O2-type structure is vital to inhibit voltage decay and hysteresis in LRLO materials. Inspired by this, Cui et al. reported that the O2-type Li_1.2_Ni_0.13_Co_0.13_Mn_0.54_O_2_ (O2-LR-NCM) with an in situ formed fluorinated cathode electrolyte interphase (CEI) on the surface from the decomposition of an all-fluorinated electrolyte during initial cycles. This effectively improved the reversibility of TM migration and surface lattice oxygen stability, resulting in minimal voltage fade [79]. All XANES spectra barely changed during the first 100 cycles for O2-LR-NCM in Fig. 6c, whereas significant reduction in TM ions and activation of new redox couples at lower voltages were observed in O3-LR-NCM. The differences are attributed to the different oxygen packing between O2-LR-NCM and O3-LR-NCM and the in situ formation of LiF CEI on electrode surface. The synergic effect of the O2-type structure and the robust CEI effectively restricted TM ions from migrating into the Li layer, prevented side reactions between electrolyte and cathodes, suppressed structural evolution from layered-to-spinel structures, and minimized irreversible oxygen loss, ultimately achieving slight voltage fade.

#### Recent Advances of the O2-Type LRLO Cathodes

Eum and co-workers’ study, constructing O2-type structure to improve the reversibility of local structural transformations, opened a new door to address the issues of voltage decay in LRLO materials. It has been considered a promising strategy to address voltage decay issue for LRLO cathodes. Researchers have explored a wide range of O2-type LRLO materials, employing various strategies to further enhance their electrochemical performance, stability, and reversibility, with a focus on suppressing voltage decay and voltage hysteresis. These strategies include: (i) optimizing ion exchange synthesis conditions to improve the transformation efficiency and structural stability, (ii) element doping to suppress irreversible TM migration and enhance structural stability, such as F doping, (iii) tailoring TM distribution to prevent phase transitions and oxygen loss, (iv) designing native vacancies to improve oxygen redox reversibility, (v) constructing hybrid O2/O3 structures to combine the advantages of both phases, such as high capacity and structural stability, and (vi) suppressing the peroxo-like O_2_^2−^ dimer formation to reduce voltage hysteresis and improve energy efficiency.

**Fig. 7:**
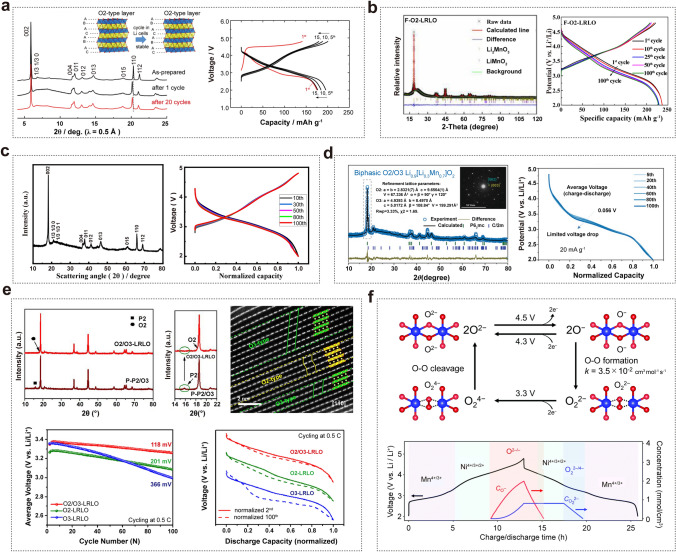
**a** Changes in SXRD patterns of O2-type Li_*x*_Li_1/4_Mn_3/4_]O_2_ during continuous cycles in Li cells and the charge/discharge curves from Ref. [80]. Reproduced with permission. Copyright 2014, Wiley–VCH. **b** XRD Rietveld refinement patterns and charge/discharge curves of Li_1.2_Ni_0.13_Co_0.13_Mn_0.54_O_2+δ−*x*_F_*x*_. Reproduced with permission from Ref. [81]. Copyright 2021, American Chemical Society. **c** XRD pattern and normalized charge/discharge curves at a current density of 200 mA g^‒1^ after 100 cycles of O2-type Li_0.78_[Li_0.24_Mn_0.76_]O_2_ nanowires. Reproduced with permission from Ref. [82]. Copyright 2020, American Chemical Society. **d** XRD refinement results of biphasic O2/O3-type Li_0.9_[Li_0.3_Mn_0.7_]O_2_ and normalized discharge curves. Reproduced with permission from Ref. [84]. Copyright 2022, Wiley–VCH. **e** XRD patterns, HRTEM images, average discharge voltage profiles, and normalized discharge profiles of biphasic O2/O3-type LRLO cathode. Reproduced with permission from Ref. [85]. Copyright 2022, Elsevier. **f** Square scheme of oxygen redox reaction in O2-Li_1.12−__y_Ni_0.17_Mn_0.71_O_2_: square scheme for polarizing oxygen redox reaction and time dependence of O^‒^ and O_2_^2‒^ concentration upon charge/discharge at C/20 calculated using the kinetic model based on the square scheme. Reproduced with permission from Ref. [86]. Copyright 2022, Royal Society of Chemistry

For example, Yabuuchi et al. prepared O2-type Li_2/3_[Li_1/6_Mn_5/6_]O_2_ materials using an ion exchange method from P2-type Na_2/3_[Li_1/6_Mn_5/6_]O_2_ precursor. This precursor features two TMO_6_ layers with partial in-plane √3a × √3a-type Li/Mn ordering (Fig. 7a**)** [80]. The transformation involved a glide of TMO_6_ layers that preserved the in-plane cation ordering after Na/Li ion exchange. Notably, in-plane cation ordering vanished after charging beyond the voltage plateaus, suggesting structural reorganizations accompanied by irreversible oxygen loss, akin to what is observed in O3-type LRLO materials. Fortunately, such detrimental phase transitions were effectively inhibited by the unusual oxygen packing in the O2-type structure. Li substitution for manganese also effectively suppressed the complicated phase transition for the P2-phase in Na cells. For example, F-doped O2-type Li_1.2_Ni_0.13_Co_0.13_Mn_0.54_O_2+*δ*−*x*_F_*x*_ leveraged the benefits of F doping and O2-type structure to suppress irreversible TM migration and enhance structural stability, curbing voltage decay (Fig. 7b**)** [81]. Shang et al. prepared O2-type Li_0.78_[Li_0.24_Mn_0.76_]O_2_ nanowires by means of ion exchange from P2-type Na_5/6_[Li_1/4_Mn_3/4_]O_2_ nanowires in a molten salt. After 100 cycles, there is no voltage decay, attributed to the O2-type structure preventing irreversible oxygen release and layered-to-spinel transition (Fig. 7c) [82]. Moreover, this material delivered an extraordinary reversible capacity of 400 mAh g^−1^ when combined with a single-layer Li_2_MnO_3_ superstructure [83].

Cao et al. designed a Co/Ni-free biphasic O2/O3-type Li_0.9_[Li_0.3_Mn_0.7_]O_2_ Li-rich layered cathode by combining 81% layered monophasic O2-type Li_2/3_[Li_2/9_Mn_7/9_]O_2_ and 19% O3-type Li[Li_1/3_Mn_2/3_]O_2_ prototypes (in Fig. 7d**)**. This configuration greatly enhanced energy density and restrained the layered/spinel phase transition, maintaining stable voltage and long-term cycle stability with 88.1% capacity retention after 500 cycles between 2.0 and 4.8 V [84]. In addition, the impact of different ion exchange methods on the structural evolution and lattice oxygen activity in O2-type Li_0.66_[Li_0.12_Ni_0.15_Mn_0.73_]O_2_ was further investigated [87]. Compared to the electrochemical ion exchange method, the chemical ion exchange method proved to be more effective in suppressing the layer-to-spinel phase transition and reducing lattice oxygen loss. Furthermore, a Li–O–Na configuration in layered Li[Na_1/3_Ru_2/3_]O_2_ was proposed by a chemical ion exchange method with Na[Na_1/3_Ru_2/3_]O_2_ as a precursor, which achieved a stable output voltage by stabilizing the oxygen redox chemistry and improving structural stability [88]. Chen et al. proposed a new O2/O3-type cathode with a homogeneous hybrid structure. This cathode exhibited lower voltage decay, improved structural stability, reduced volume change and minimized irreversible oxygen loss compared to pure O2-type and O3-type phases (Fig. 7e) [85]. Additionally, other strategies were explored to enhance the voltage/capacity stability and improve the reversibility of local structural transformations in O2-type LRLO materials, such as comparing different ion exchange methods, tailoring TM distribution, and designing native vacancies [89–91]. In 2018, Yamada et al. investigated cobalt-free O2-type *x*Li[Li_1/4_Mn_3/4_]O_2_-(1−*x*)Li[Mn_2/3_Ni_1/3_]O_2_ materials, which exhibited slower voltage fading than O3-type LRLO upon cycling, highlighting that the inclusion of expensive cobalt was not essential for achieving high capacities in O2-type materials, but it did contribute to improving initial coulombic efficiency [92]. They also studied O2-type Li_1.22−*x*_Ru_0.78_O_2_ cathode with strong Ru‒O covalent bonds, which helped suppress the lattice oxygen activity and improve structural integrity, resulting in negligible voltage hysteresis [93]. In 2022, Yamada et al. synthesized O2-Li_1.12−*y*_Ni_0.17_Mn_0.71_O_2_ using P2-Na_0.71_[Li_0.12_Ni_0.17_Mn_0.71_]O_2_ as a precursor. This material exhibited coexisting nonpolarizing O^−^ ↔ O^2−^ (4.4 V *vs.* Li/Li^+^) and polarizing O_2_^2−^  → O^2−^ (3.3 V *vs.* Li/Li^+^), which kinetically competed [86]. The oxygen redox reaction was described as a square scheme in Fig. 7f, involving bond-forming 2O^−^ → O_2_^2−^ and bond-cleaving O_4_^2−^ → 2O^2−^ processes, where suppressing the formation of peroxo-like O_2_^2−^ dimer played a vital role in achieving nonpolarizing and energy-efficient oxygen redox reactions because the kinetic formation of O_2_^2−^ was identified as the origin of a voltage hysteresis.

The above studies confirmed that O2-type LRLO materials exhibited much weaker voltage decay than their O3-type counterparts. These studies provide a solid foundation for the design of high-performance O2-type LRLO materials. It identifies areas where further improvements are needed, such as optimizing ion exchange methods, exploring new structural designs, and understanding the long-term degradation mechanisms. Although O2-type structure avoids the inherent defects of the O3-type structure, it still exhibits a certain degree of voltage decay. Therefore, other factors on voltage decay in O2-type structure need to further deeply explored.

**Fig. 8:**
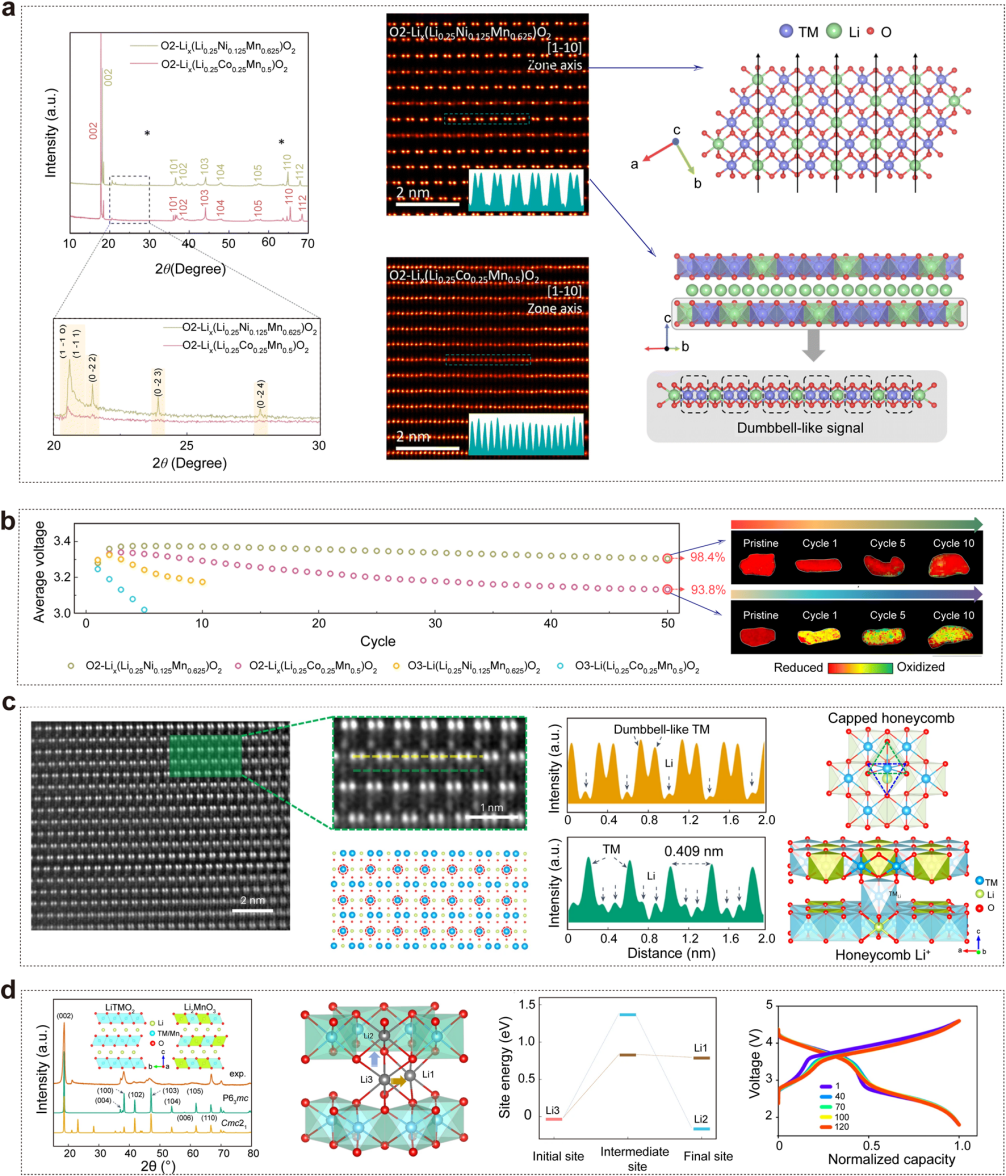
**a** Comparison of the in-plane cation ordering in O2-Li_*x*_(Li_0.25_Ni_0.125_Mn_0.625_)O_2_ and O2-Li_*x*_(Li_0.25_Co_0.25_Mn_0.5_)O_2_ electrodes: HRPD patterns, HAADF-STEM images, and top view of the atomic arrangement in a TM layer. **b** Comparison of average discharge voltages and spatial distribution of oxygen redox degree. Reproduced with permission from Ref. [14]. Copyright 2023, Royal Society of Chemistry. **c** Structure of Li_1.1_(Ni_0.21_Mn_0.65_Al_0.04_)O_2_: STEM-HAADF image along the [110] zone axis and HADDF in-line profile, sectional view of the structure with TMLi atoms located just next to the honeycomb, and the schematic diagram of the capped-honeycomb local structure. **d** Experimental XRD data along with calculated XRD patterns of O2-type layered LiTMO_2_ and Li_2_MnO_3_ phases, migration barriers and normalized voltage/capacity profiles in full cells with a graphite anode. Reproduced with permission from Ref. [13]. Copyright 2023, Nature Publishing Group

O2-type LRLO materials still exhibited a partially disordered structure even after extended cycles, implying that additional factors trigger the irreversible TM migration. Eum et al. explored the connection between superstructure ordering and the tendency for irreversible TM migration, aiming to identify the cause of voltage decay in O2-type LRLO materials [14]. They emphasized the significant impact of substituent species and the initial cation configuration in TM layers on the extent of voltage decay. Moreover, the absence of superstructure ordering in the pristine material was found to accelerate irreversible TM migration, resulting in quicker voltage decay, faster electrochemical degradation, and an asymmetric oxygen redox transition. The O2-Li_*x*_(Li_0.25_Ni_0.125_Mn_0.625_)O_2_ (LLNMO) presented obvious superstructure peaks in Fig. 8a, indicative of the typical honeycomb structure, whereas these peaks were barely observed in O2-Li_*x*_(Li_0.25_Co_0.25_Mn_0.5_)O_2_ (LLCMO). This demonstrated that the Ni^2+^ substitution effectively preserved the in-plane honeycomb orderings, while Co^3+^ disarranged the in-plane cation ordering. The distinct in-plane configurations were directly visualized through spherical-aberration-corrected scanning transmission electron microscopy (Cs-STEM) images, where a clear dumbbell-like signal was observed in the monolayers of LLNMO electrode, in contrast to the randomly distributed TM ions in the LLCMO electrode.

The average discharge voltages O2-type electrodes are significantly more stable than those of O3-type counterparts (Fig. 8b**)**, aligning with previous reports [81, 84, 85, 94]. Nevertheless, the voltage decay of the LLCMO electrode was observed to be slightly faster than that of the LLNMO. In addition, cycle-dependent STXM spectra of the O K-edge revealed notable differences in oxygen redox behaviors between the two electrodes, with the LLNMO electrode demonstrating superior reversibility post-cycling. These observations underscore the critical role of the honeycomb structure in mitigating irreversible TM migration in O2-type LRLO cathode materials.

Luo et al. made significant strides in enhancing the stability of the honeycomb structure within the Li_2_MnO_3_ component to tackle the persistent issue of voltage decay by constructing capped-honeycomb structure. They developed a Co-free Li_1.1_(Ni_0.21_Mn_0.65_Al_0.04_)O_2_ (CH-LMR) cathode material with negligible voltage decay, synthesized by an ion exchange method using a P2-type Na/LiTMO_2_ as a precursor [13]. As shown in Fig. 8c, d, the CH-LMR cathode displayed an O2-type structure, comprising layered hexagonal (*P*6_3_*mc*) LiTMO_2_ and orthorhombic (*Cmc*2_1_) Li_2_MnO_3_ components. The STEM-HADDF image exhibited TM ions partially occupying the interlayer Li_3_ sites, either directly below or above the Li atoms in the honeycomb structure of the Li_2_MnO_3_ phase, forming an ordered substitution of one TM for every three Li ions. The periodicity of this repeating unit [Li_2/3_Mn_1/3_] is the same as that of [Li_1/3_Mn_2/3_] in the TM layers. Unlike conventional honeycomb structures in LRLO cathodes where O atoms in the LiO_6_ honeycomb are unstable at high voltages, leading to irreversible O_2_ release, the capped-honeycomb structure with TMLi_3_ plays a crucial role in inhibiting cation migrations, oxygen release, and phase transitions by localizing the O 2*p* lone-pair state [18]. First-principles calculations further demonstrated that the TM-pinned honeycomb structure with TM occupying the Li_3_ site played a key role in stabilizing the lattice oxygen. This study opens a new avenue to inhibit the voltage decay in LRLO cathode materials, which will accelerate the commercial application of the next-generation high-energy cathode materials for practical LIBs.

## Conclusions and Perspectives

In conclusion, the issue of voltage decay remains a significant barrier to the practical application of LRLO materials. It is widely recognized that the irreversible TM migration is the origin of voltage decay, exacerbating structural evolution including phase transitions from layered phase to spinel phase or even rock salt phase and the formation of microscopic defects, such as nanovoid and densification. These often aggravate the irreversible oxygen redox reactions, resulting in oxygen release and the formation of oxygen vacancies, typically accompanied by TM ions reduction and dissolution. To address this problem, researchers have proposed various strategies including element doping, surface coating, morphological design, structure design, and modulation of binder and electrolyte. However, for O3-type LRLO materials, TM ion migration from the adjacent tetrahedral sites to the neighboring adjacent octahedral Li sites is the thermodynamic preference and irreversibly, making these modification methods only partially effective and insufficient for commercial applications.

Research into O2-type layered oxide materials has demonstrated that the O2-type configuration significantly outperforms the O3-type structure in suppressing voltage decay. For O2-type LRLO materials, the substantial electrostatic repulsion between cations in face-shared octahedral sites inhibits TM migrations from intermediate tetrahedral sites to adjacent octahedral Li sites, facilitating the return of migrated TM ions to the octahedral site of the TM layers during the discharge process. Despite these advancements, reported O2-type LRLO materials still exhibit partially disordered structures with extended cycles, indicating the presence of additional factors that trigger irreversible TM migrations. Moreover, other electrochemical performances, particularly long-term cycle performance, have not been fully optimized. Therefore, relentless efforts are necessary to design and develop high-performance O2-type LRLO materials. In the pursuit of practical applications, integrating multidimensional strategies is crucial to meet multiple requirements and enhance the overall performance of O2-type LRLO materials. The design of high-performance O2-type LRLO materials should focus on the following four aspects to boost the commercialization process:

1. P2-type precursor design
  As metastable materials, O2-type LRLO materials cannot be achieved by high-temperature lithiation reactions but only by ion exchange method using P2-type sodium layered oxides as precursors. Therefore, the structure and composition of P2-type precursors play a crucial role in influencing the Li^+^ local environment in the Li layer and the distribution of various elements in the TM layer of corresponding O2-type LRLO materials. We believe that factors such as the Na^+^/vacancy arrangement, component, particle size, morphology, and sodium content in P2-type precursors can significantly impact the voltage stability and structural stability of O2-type LRLO materials. However, there is a lack of systematic research in this area. Therefore, we encourage researchers to explore along this line.
2. O2-type preparation conditions
  O2-type LRLO materials were synthesized by Li/Na ion exchange methods. The P2-type sodium layered oxides precursors need to be dispersed in an organic solution or molten salts containing compounds with lithium ions, such as LiCl, LiBr, and LiNO_3_. It is crucial to highlight that ion exchange process cannot be conducted in an aqueous solution, because the precursors typically undergo an irreversible phase transition, or even structural collapse, when exposed to water. Optimizing the preparation conditions is vital for obtaining a pure and stable O2-type structure. The types of organic solvents, the concentration and types of salts, reaction time, and reaction temperature significantly impacts the transformation efficiency and structural stability of O2-type LRLO materials. For example, when the reaction temperature is excessive, the O2-type structure will transform into a O3-type structure or spinel phase. To be more suitable for industrial amplification, we believe that conducting the ion exchange process in an organic solution under reflux condition shows great promise.
3. Bulk structure design
  The design of the bulk structure is pivotal. Studies reveal that the presence of a superstructure configuration corresponding to the honeycomb structure in O2-type LRLO materials plays a vital role in inhibiting the irreversible migration of TM ions, consequently resulting in a slight voltage decay. Moreover, enhancing the stability of the superstructure configuration could further suppress irreversible TM migrations. We believe that the cation arrangements in the TM layer and introduction of pillar elements in alkali metal layer in P2-type precursor directly affect the stability of the bulk structure for O2-type LRLO materials. However, research in this direction is currently limited, warranting more attention to the influence of doping elements on O2-type bulk structure, including types of doping elements, single- or multi-element doping strategy, dual-site doping, and the solubility or compatibility of doping elements in LRLO materials. It is well-known that achieving doping in the Li layer for O3-type LRLO materials is difficult, while it is relatively easy for O2-type LRLO materials. For example, we first prepare Ca-pillared, Mg-pillared, or Zn-pillared P2-type precursors, and then obtain the corresponding Ca-pillared, Mg-pillared, or Zn-pillared O2-type LRLO materials through ion exchange methods.
4. Surface structure design
  Compared with the bulk structure, the surface structure with much weaker bonds is easier to be corroded by electrolytes during charging/discharging process. For example, the surface lattice oxygen contacted with electrolytes is prone to release as O_2_, especially in O2-trpe LRLO materials with more defects, making surface modifications challenging. Yet, obtaining a stable surface structure is indispensable. Exploring more effective modification methods and new techniques for O2-type structures is encouraged, considering practical aspects such as coating material cost, manufacturing cost, and operational complexity.

Overall, we anticipate that this review will provide valuable insights into the design of high-performance LRLO materials. Through persistent research and collaborative initiatives, we are optimistic that high-performance LRLO cathode materials will soon become a practical reality for next-generation lithium-ion batteries.
